# Audit on Insomnia Management in In-Patient Psychiatric Unit

**DOI:** 10.1192/bjo.2026.11846

**Published:** 2026-06-30

**Authors:** Bhoomika Soravanahally Anandakumar, Mohammed Elsankary, Anisha Malli, Zoe Tebbs

**Affiliations:** Hampshire and Isle of Wight Foundation Trust, Southampton, United Kingdom

## Abstract

**Aims:**

- Evaluating the current practice for management of insomnia in inpatient settings.

- Assessment of the current use of CBT- Insomnia for the treatment of sleep disorders in inpatient settings.

- Comparing the current practice with the NICE guidelines.

**Methods:**

A retrospective review of electronic medical records of 45 patients who were currently admitted in the inpatient unit over a period of two weeks of data collection.

For each patient, documentation was examined to determine:

• Whether insomnia was reported or observed

• Whether sleep difficulties were noted in clinical records

• Whether nonpharmacological interventions (e.g., sleep hygiene) were offered

• Whether behavioural therapy (CBTI), which is considered the first line of treatment.

treatment for chronic insomnia was offered or signposted

• Whether hypnotic medication was prescribed, and if so:

Type of medication

Documentation of indication

Presence of review or stop dates

**Results:**

Out of the 45 current in-patients audited during the initial assessment of electronic records, 26 (58%) complained of sleeping difficulties; an additional 4 patients were observed to have sleeping difficulties according to the records. This makes the totalprevalence of insomnia 30 (66.6%). Of these patients, 23 (76.6%) had proper documentation of insomnia in their notes. As management, non-pharmacological interventions such as sleep hygiene discussion were done in 11 out of 30 (36.6%), and no patients (0%) were offered or signposted towards CBT-I. Pharmacological interventions of offering hypnotic medications were offered to 26 out of 30 (86.6%). The hypnotic medications offered included Z-drugs and/or in combination with benzodiazepine and antihistamines. None of the patients (0%) adhered to all criteria of NICE guidelines and Local Trust Policy (classifying insomnia according to duration of symptoms, trial of non-pharmacological intervention before using medications, clear indication documented before starting hypnotics, periodic review and documentation of stop date for said medications)

**Conclusion:**

This audit highlights the need for a structured and consistent approach to managing insomnia across the hospital. Establishing a simple, standardized care pathway–prioritizing non-pharmacological strategies and applying a cautious, clearly justified approach to hypnotic prescribing–has the potential to enhance patient safety, minimize the risk of hypnotic dependence, and promote higher-quality clinical care. Ensuring insomnia is managed systematically and in line with NICE recommendations will support more effective and sustainable outcomes for our patients.

